# Characterization of Flucloxacillin-specific CD8+ T-cells in a mouse model

**DOI:** 10.1186/2045-7022-4-S3-P41

**Published:** 2014-07-18

**Authors:** Ryan Nattrass, Lee Faulkner, Roz Jenkins, Jean Nicolas, Marc Vocanson, Aurore Rozieres, Daniel Antoine, Anja kipar, Kevin Park, Dean Naisbitt

**Affiliations:** 1 Liverpool University, MRC Centre for Drug Safety Science, UK; 2 Lyon 1 University, UFR Lyon Sud and Hospices Civils de Lyon, Deparment of Clinical Immunology and Allergy, France

## 

Drug-induced liver injury (DILI) involving the adaptive immune system is a serious complication in both patient treatment and drug development. Flucloxacillin, a -lactam antibiotic effective against -lactamase-producing bacteria, is a common cause of immunological DILI through cholestasis. We have recently isolated CD8+ flucloxacillin-specific T-cells from patients with DILI, which indicates that T-cells participate in the pathogenesis of the disease. There are currently no animal models of DILI where the adaptive immune system has shown to damage liver, and therefore, it is difficult to explore the mechanistic basis of the tissue injury. Thus, the aim of this study was to use C57/Bl6 CD4 deficient mice with a mutation in the gene encoding for MHC class II molecules to explore the immunogenicity of flucloxacillin and whether flucloxacillin-responsive CD8+ T-cells damage hepatocytes. In initial experiments sensitization was achieved through epicutaneous application (1g/ml; in 70% DMSO; 50L). Proliferation, IFN- and granzyme-B secretion from draining lymph node cells was measured ex vivo following treatment with flucloxacillin. Flucloxacillin-specific CD8+ T-cell mediated killing of hepatocytes was measured using the ApoTox-Glo kit (Promega, UK). In separate experiments, flucloxacillin-specific T-cells were forced to migrate to the mesenteric lymph nodes using retinoic acid, prior to administration of oral flucloxacillin for 10 days, followed by analysis of plasma biomarkers of liver injury. CD8+ T-cells from draining lymph nodes of flucloxacillin-treated mice proliferated in a concentration-dependent manner following ex vivo secondary stimulation. The proliferative response was associated with IFN- and granzyme-B secretion. In contrast, proliferative responses and cytokine secretion was not detected with cells from vehicle control mice. Flucloxacillin-specific hepatocyte toxicity and apoptosis was observed when CD8+ T-cells were cultured with dendritic cells and flucloxacillin for 24h, washed and transferred to the hepatocyte cultures. Flucloxacillin sensitization followed by 10 day oral exposure resulted in marked gall bladder swelling and mild elevations in ALT. Other plasma biomarkers of liver damage were negative. In conclusion, these data show successful sensitization of mice against flucloxacillin. Thus, the C57/Bl6 CD4 deficient mouse represents a promising model to study the role of the adaptive immune system in DILI.

